# Impaired episodic verbal memory recall after 1 week and elevated forgetting in children after mild traumatic brain injury – results from a short-term longitudinal study

**DOI:** 10.3389/fpsyg.2024.1359566

**Published:** 2024-06-03

**Authors:** Karen Lidzba, Zainab Afridi, Fabrizio Romano, Kevin Wingeier, Sandra Bigi, Martina Studer

**Affiliations:** ^1^Division of Neuropediatrics, Development and Rehabilitation, Department of Paediatrics, Inselspital, Bern University Hospital, University of Bern, Bern, Switzerland; ^2^Division of Paediatric Emergency Medicine, Department of Paediatrics, Inselspital, Bern University Hospital, University of Bern, Bern, Switzerland; ^3^Department of Psychosomatics and Psychiatry, University Children’s Hospital Zurich, Zürich, Switzerland; ^4^Institute for Social and Preventive Medicine, University of Bern, Bern, Switzerland; ^5^Division of Pediatric Neurology, Department of Pediatrics, Children’s Hospital of Central Switzerland, Lucerne, Switzerland; ^6^Department of Pediatric Neurology and Developmental Medicine, University Children’s Hospital Basel (UKBB), Basel, Switzerland; ^7^Department of Clinical Research, University of Basel, Basel, Switzerland; ^8^Department of Neurology, Inselspital, Bern University Hospital, University of Bern, Bern, Switzerland; ^9^Department of Psychology, University of Basel, Basel, Switzerland

**Keywords:** mild traumatic brain injury, accelerated long-term forgetting, delayed episodic memory recall, memory consolidation, executive functions

## Abstract

**Objective:**

There is preliminary evidence that children after traumatic brain injury (TBI) have accelerated long-term forgetting (ALF), i.e., an adequate learning and memory performance in standardized memory tests, but an excessive rate of forgetting over delays of days or weeks. The main aim of this study was to investigate episodic memory performance, including delayed retrieval 1 week after learning, in children after mild TBI (mTBI).

**Methods:**

This prospective study with two time-points (T1: 1 week after injury and T2: 3–6 months after injury), included data of 64 children after mTBI and 57 healthy control children aged between 8 and 16 years. We assessed episodic learning and memory using an auditory word learning test and compared executive functions (interference control, working memory, semantic fluency and flexibility) and divided attention between groups. We explored correlations between memory performance and executive functions. Furthermore, we examined predictive factors for delayed memory retrieval 1 week after learning as well as for forgetting over time.

**Results:**

Compared to healthy controls, patients showed an impaired delayed recall and recognition performance 3–6 months after injury. Executive functions, but not divided attention, were reduced in children after mTBI. Furthermore, parents rated episodic memory as impaired 3–6 months after injury. Additionally, verbal learning and group, but not executive functions, were predictive for delayed recall performance at both time-points, whereas forgetting was predicted by group.

**Discussion:**

Delayed recall and forgetting over time were significantly different between groups, both post-acutely and in the chronic phase after pediatric mTBI, even in a very mildly injured patient sample. Delayed memory performance should be included in clinical evaluations of episodic memory and further research is needed to understand the mechanisms of ALF.

## 1 Introduction

Traumatic brain injury (TBI) is associated with cognitive, emotional, behavioral and social impairments in children and adolescents (Li and Liu, 2013; Ryan et al., 2014; Moran et al., 2016). The severity of TBI is defined according to the Glasgow coma scale (GCS), which has three levels: Mild TBI (GCS 15–13), moderate TBI (GCS 12–8) and severe TBI (GCS ≤8) (Teasdale and Jennett, 1974). The worldwide incidence of pediatric TBI varies considerably; reported annual rates range between 47 and 280 per 100,000 children (Dewan et al., 2016). Given that about 90% of all such injuries are classified as mild TBI (mTBI) or concussion, mTBI is one of the leading consequences of injury in childhood (Hon et al., 2019).

Several studies have observed that children after moderate and severe TBI primarily have impairments in processing speed, attentional and executive functions, and in episodic memory (Anderson and Catroppa, 2005; Catroppa and Anderson, 2007; Beauchamp et al., 2011). There is a dose–response relationship regarding the severity of trauma and the associated neurocognitive impairments (Babikian et al., 2015). However, data on neuropsychological outcome after pediatric mTBI are less consistent: Systematic reviews have concluded that children after mTBI have very few, if any, long-term neurocognitive impairments (Satz et al., 1997; Babikian and Asarnow, 2009).

Only a few studies have pointed to episodic memory impairments in adults after mTBI (Wammes et al., 2017; Fortier-Lebel et al., 2021) and recent studies concluded that memory complaints may not be associated with performance in memory tests after mTBI (Anderson, 2021; Rioux et al., 2022). Similarly, parents of children who have sustained mTBI report memory problems (Waldmeier-Wilhelm et al., 2019), although studies rarely report the performance of specific neuropsychological testing to identify episodic memory impairments (Chadwick et al., 2021). A recent study investigated the trajectory of cognitive functions depending on the severity of post-concussive symptoms in pediatric mTBI patients. The authors concluded that verbal memory outcome was the only cognitive domain that was significantly reduced in symptomatic and asymptomatic mTBI patients compared to healthy controls, even in the early chronic phase 4 months after injury (Robertson-Benta et al., 2023).

Episodic memory allows us to learn, store and recall our daily personal experiences, supported by a distributed cortical and subcortical network, including the prefrontal, temporal and parietal cortex (Dickerson and Eichenbaum, 2010). Deficits in episodic memory are usually detected using standardized memory tests in which memory recall performance is tested about 20–30 min after learning. However, there are studies implying that some patients showing a normal memory recall in common standardized tests have accelerated long-term forgetting (ALF) over time, a recently identified memory phenomenon that was first described in adult patients with epilepsy (Elliott et al., 2014). Thus, these patients experience a faster than normal loss of newly learned information over extended periods, which is not detectable in standardized memory assessments. So far, ALF has been identified in adult patients with epilepsy (Mameniskiene et al., 2020), after stroke (Geurts et al., 2019; Lammers et al., 2022), and in patients with pre-symptomatic Alzheimer disease (Weston et al., 2018; Rodini et al., 2022). To date, only a few studies have investigated ALF in childhood, most of which were conducted in children with epilepsy (Stähli et al., 2022). Given that most studies so far have focused on epilepsy patients, it was assumed that ALF is related to the presence of seizures. However, there is preliminary evidence that ALF also occurs in non-epileptic pediatric patients, for example, in children after severe TBI (Lah et al., 2017). It is not yet clear whether children after mTBI may also exhibit ALF.

Furthermore, so far, it is unclear whether ALF represents an impairment of encoding, retrieval or consolidation. Given that patients with ALF show a relatively intact memory performance after short delays, but an impairment after longer delays, it was concluded that ALF does not represent an acquisition impairment, but rather a consolidation impairment (Blake et al., 2000; Hoefeijzers et al., 2013; Mameniskiene et al., 2020; Studer et al., 2024). To clarify whether ALF reflects an impairment of memory consolidation or of memory retrieval, it is essential to measure recognition. Since recognition tests demand less cognitive effort than free recall tests, it was suggested that reduced free recall after 1 week with normal cued recognition performance could illustrate a retrieval impairment, as shown in children after TBI (Lah et al., 2017) and in children with idiopathic generalized epilepsy (Grayson-Collins et al., 2019), possibly associated with subtle executive function impairments (Grayson-Collins et al., 2019; Mameniskiene et al., 2020). However, if verbal recognition and recall after 1 week is impaired, as seen in children with idiopathic generalized epilepsy (Gascoigne et al., 2012) and in children with 22q11.2 deletion syndrome (Maeder et al., 2020), ALF could be interpreted as a consolidation problem. So far, few studies in children have investigated delayed recall and recognition performance, although such studies would be necessary to determine whether ALF is an impairment of retrieval, consolidation or both (Stähli et al., 2022).

The aim of this study was threefold: First, we wanted to investigate episodic memory performance (recall and recognition) at two time-points: Post-acutely at T1 (1 week after injury) and in the early chronic phase at T2 (3–6 months after injury). We hypothesized that, post-acutely, children after mTBI will exhibit reduced delayed memory retrieval 30 min and 1 week after learning, but that in the chronic phase after mTBI, episodic memory retrieval (30 min and 1 week after learning) will be comparable to that of healthy controls. Second, we aimed to explore whether episodic memory performance correlates with executive functions as well as with parents’ rating of memory in everyday life. Third, we wanted to identify the factors that predicted delayed verbal recall performance after 1 week as well as forgetting at both time-points.

## 2 Methods

The study was approved by the Ethics Committee of the University Children’s Hospital in Bern and by the Bernese Cantonal Ethics Committee (number: 2020–00596). All caregivers (as well as adolescents aged 14 years and older) provided informed consent before participation, consistent with the Code of Ethics of the World Medical Association (Declaration of Helsinki).

### 2.1 Design

This longitudinal study with two time-points within 3–6 months after a mTBI is part of the influence of episodic long-term memory on participation after pediatric mild traumatic brain injury project, a single-center longitudinal observational study.

### 2.2 Participants and sample attrition

For this study, 64 children after a mTBI and 57 healthy control children between the ages of 8 and 16 years were recruited between June 2020 and December 2021. The recruitment of the mTBI patients took place in the Pediatric Emergency Department (PED) of the University Children’s Hospital Bern (Inselspital). Healthy control participants were recruited through flyers, social media, sports clubs, or by the study team. All healthy controls were screened to ensure they fulfilled the inclusion criteria. The general inclusion criteria were: age between 8 and 16 years, German-speaking, informed consent/assent of the child/adolescent or informed consent of the parents of children younger than 14 years, and normal schooling following the regular curriculum. Specific inclusion criteria for patients after mTBI were defined according to the World Health Organization criteria (Carroll et al., 2004): GCS of 13–15 after the injury; a traumatically induced physiological disruption of brain functioning manifesting in at least one of the following: loss of consciousness (<30 min), post-traumatic amnesia (<24 h), or mental changes (confusion or disorientation) at the injury time-points. The patients with mTBI were observed in the hospital for some hours. General exclusion criteria were: intellectual disability (IQ < 70), severe language disorders (i.e., dysphasia, aphasia, language impairment), insufficient knowledge of the German language, autistic spectrum disorder, neurological condition in medical records, previous brain injuries, intake of psychotropic medication, severe visual impairments not correctable by glasses, or severe hearing problems not correctable by hearing aids. Maternal education was included as a proxy for socioeconomic status (SES); it was defined as the mother’s years of education at the time of the child’s neuropsychological assessment (schooling and formal education). Pre-injury learning impairments [learning problems, dyslexia or attention deficit hyperactivity disorder (ADHD)] as well as pre-injury mood impairments (depression, and anxiety) and previous mTBI were reported by parents at T1 and concatenated to the variable pre-existing problems. During our study period, 236 children treated in the PED were diagnosed with mTBI. Of these, 172 did not participate due to lack of time (7%), long distance from home (18%), foreign language (7%), health problems (12%), no contact with the study team at the PED (10%), no interest in the study (17%), or other reasons (27%). As illustrated in Table 1, we included 64 patients after mTBI and 57 healthy controls. Six patients underwent initial magnetic resonance imaging but only one showed abnormalities (minor bleeding temporal left and minor bleeding bifrontal). Patients who participated were comparable with those who did not regarding age (*M* participants (*SD*) = 10.81 (2.26) years; *M* non-participants (*SD*) = 11.19 (2.28) years, *t*(234) = −1.14, *p* = 0.256, *d* = −0.167) and sex [male participants: 35 (54.68%)]; male non-participants: 104 (60.12%; *X^2^* (1) = 0.505, *p* = 0.477). Furthermore, the number of weeks between T1 and T2 were comparable between patients (*M* = 19.34, *SD* = 4.17) and healthy controls (*M* = 17.87, SD = 8.61; *t*(106) = 1.15, *p* = 0.127, *d* = 0.22).

**Table 1:** Demographic and clinical variables.

Sample size	Healthy controls	mTBI patients	Test of significance *(df)*	*p*-value	Effect size
T1, *n* (% male)	57 (40%)	64 (55%)	*X^2^*(1) = 2.48	0.115	*ɸ* = 0.14
T2, *n* (% male)	52 (42%)	56 (55%)	*X^2^*(1) = 1.84	0.245	*ɸ = 0*.13
Days between T1 and T2, M (SD)	127.23 (61)	137.29 (29.19)	*t*(106) = 1.1	0.136	*d* = 0.21
Age in years at injury, *M (SD)*	11.75 (2.39)	10.73 (2.06)	*t*(119) = −2.52	0.013*	*d* = −0.46
SES (Years of maternal education), *M (SD)*	14.61 (2.56)	13.75 (2.67)	*t*(119) = −1.81	0.072	*d* = −0.33
IQ WISC-V, *M (SD)*	112.55 (11.87)	107.22 (12.33)	*t*(104) = −2.26	0.026	*d* = −0.44
GAI WISC-V, *M (SD)*	111.49 (10.98)	108.82 (11.97)	*t*(104) = −1.20	0.235	*d* = −0.23
CPI WISC-V, *M (SD)*	111.94 (13.47)	104.05 (13.74)	*t*(105) = −3.00	0.003	*d* = −0.58
Previous mTBI, *n* (%)	2 (4%)	9 (14%)	*X^2^*(1) = 4.06	0.044*	*ɸ* = −0.18
Pre-injury diagnosis, *n* (%)^b^	2 (3.5%)	6 (9.4%)	*X^2^*(1) = 1.68	0.195	*ɸ* = −0.12
Learning problems	0	2	–	–	–
Dyslexia	2	4	–	–	–
ADHD	0	2^a^	–	–	–
Depression	0	0	–	–	–
Anxiety	0	0	–	–	–
Injury characteristics					
GCS at injury, *M (SD)*		14.9 (0.43)			
Intracranial injury, *n* (%)		1 (1.56%)			
Loss of consciousness, *n* (%)		16 (25%)			
Duration loss of consciousness in sec, *M* (SD)		124.6 (109.8)			
Post-traumatic amnesia, *n* (%)		34 (53%)			
Cause of injury					
Fall, *n* (%)		54 (84%)			
Blow, *n* (%)		10 (16%)			

^a^Both of these children had as well dyslexia.^b^All pre-injury diagnoses were reported by parents.ADHD, attention deficit hyperactivity disorder; CPI, Cognitive Proficiency Index; GAI, General Ability Index; IQ, Intelligence Quotient; mTBI, mild traumatic brain injury; SES, Socioeconomic status; WISC-V, Wechsler Intelligence Scale for Children fifth edition.*p < 0.05.

### 2.3 Procedure

This study included data from the time-points T0 (directly after the mTBI), T1 (1 week after the injury) and T2 (3–6 months after the injury). At T0, a brief cognitive and neurological screening was performed, and post-concussion symptoms reported by parents were recorded. At T1 and T2, neuropsychological testing sessions were held in the University Children’s Hospital in Bern or at the family home. The examiners were neuropsychologists or trained psychology and medical students, supervised by a certified neuropsychologist (M.S.). Participants were compensated with a media voucher worth 20 CHF.

### 2.4 Assessments and materials

*Clinical and cognitive screening in the PED*: We created a clinical–neurological screening procedure to evaluate children after mTBI in the PED. This screening assessed the following injury-related variables: cause of injury, duration of loss of consciousness, amnesia, vomiting, and GCS upon arrival at the hospital. In addition, post-concussive symptoms were rated by parents. Episodic learning and memory performance was screened using a 12-word-long word list, which was learned over three runs, and children had to freely recall these words 10 min later. For this study, we included all injury-related variables and recall performance from memory screening.

*Neuropsychological testing*: With the exception of the verbal learning and memory test scores, all raw test scores were transformed into age-corrected standard scores (SS) or T-values (T), as required by the respective test manual. The following cognitive functions were assessed in this study:

*Intellectual functioning* was measured at T2 using the Full-Scale IQ (FSIQ) (Wechsler, 2014), consisting of the first 10 Wechsler Intelligence Scale for Children® Fifth Edition (WISC-V) subtests: block design, similarities, matrix reasoning, digit span, coding, vocabulary, and figure weights, visual puzzles, picture span and symbol search. Besides FSIQ, we included the General Ability Index (GAI, consisting of the subtests similarities, vocabulary, block design, matrix reasoning and figure weights), the Cognitive Proficiency Index (CPI, consisting of the subtests digit span, picture span, coding and symbol search) as well as Working Memory Index (consisting of the subtests digit span and picture span).

*Verbal learning and memory performance* was assessed at T1 and T2 (parallel form) using a newly created auditory verbal learning and memory test [WoMBAT=Wortlisten Merken Behalten Abrufen Test, the test will be available online in fall 2024]. This test is based on the Rey Auditory Verbal Learning Test [RAVLT (Rey, 1958)], adapted for German-speaking children, and was recently used in other studies by our group (Studer et al., 2023). Children had to learn a list of 17 words over four learning runs. After each run, children were asked to recall all the words they remembered. After learning, a second list (interference list) of 17 words was read to the children. Again, children were asked to recall all the words they remembered from the second list. After listening to the interference list, children were asked to immediately recall all the words they remembered from the first word list and were told to memorize these words because they would have to remember them later on. Further free recalls of the first word list were assessed 30 min and 1 week after learning. Furthermore, 30 min after learning, recognition performance was tested with a list of 50 words containing all 17 words from the first list, all 17 words from the interference list and 16 more words with similar semantic meanings. Participants had to say ‘yes’ if a word stemmed from the first word list and ‘no’ if it did not. Testing of verbal recall 1 week after learning (1 week after T1 and T2, respectively) was conducted over the phone, as described by other groups (Gascoigne et al., 2019; Grayson-Collins et al., 2019), without informing participants in advance that they would have to remember the words a week later. Dependent variables for this study were verbal learning (sum of the words recalled over four learning runs, maximum possible raw score 68), immediate recall, free recall, and recognition performance (correct minus false-positive answers) 30 min after learning, and free recall and recognition performance (correct minus false-positive answers) 1 week after learning. Recall and recognition performance had a maximum possible raw score 17. We calculated the percentage of words forgotten between 30-min recall and recall after 1 week (recall loss after 1 week = 100*(recall 30 min after learning − recall after 1 week)/30-min recall), defined as *recall loss*.

*Executive functions* are an umbrella term that covers several different processes – we analyzed the core functions *working memory, interference control* and *flexibility*, as well as *verbal fluency*. *Working memory* was tested using the subtests digit span and picture span of the WISC-V (Wechsler, 2014); the dependent variable was working memory index. *Interference control* was tested using the interference condition of the Stroop [Delis Kaplan Executive Function System (D-KEFS) (Delis et al., 2001)]; the dependent variable was time to completion. Additionally, *flexibility* was tested using the condition furniture-name-switch at T1 and vegetables-musical instruments at T2 from the Verbal Fluency task [D-KEFS (Delis et al., 2001)]. *Verbal fluency* was tested with Category Fluency using the categories animals and boys’ names at T1 and clothes and girls’ names at T2. The dependent variable for all fluency tasks was the number of correct words.

*Divided attention* was measured using the subtest divided attention from the testing battery for attentional performance (TAP) (Zimmermann and Fimm, 2012). The dependent variable was the number of omissions (defined as the number of targets participants did not respond to although they should have).

*Parents’ rating of cognition in everyday life* was measured at T1 and T2 using the Kopkji questionnaire (Gleissner et al., 2006), a standardized parental questionnaire to assess neurocognitive problems in daily life of children between 6 and 16 years. The frequency of observed behavior is rated from 1 (never) to 4 (always); higher values mean a worse rating. For this study, we included items of the memory subscale (maximum possible raw score 60).

Study data were collected and managed using REDCap (Research Electronic Data Capture) tools (Harris et al., 2009) hosted at ARTORG, Center for Biomedical Engineering Research at the University of Bern, Switzerland.

### 2.5 Statistical analysis

Statistical analyses were performed using SPSS version 28 (IBM Corp. Released 2017. Armonk, NY: U.S.A.). Statistical significance was defined as *p* < 0.05; however, in the case of multiple comparisons, we applied the Bonferroni correction with a modified *p*-value. Effect size is reported as Cramer *V, r*, or partial *η^2^* values. If the assumption of sphericity was violated, we report the Huynh-Feldt corrected degrees of freedom. Before running our analysis, we analyzed group differences regarding demographics (the variables age, SES and FSIQ, GAI and CPI were compared by using independent *t*-tests; Pearson chi-square analysis was performed to examine group difference related to sex). Given that age was significantly different between groups, we controlled for age in all analyses. Although FSIQ also differed significantly between patients and healthy controls, we did not control for it because there was no significant difference regarding the GAI, pointing to similar verbal and visual-figural skills. Thus, the significant group difference in FSIQ was driven by the highly significant difference in the subscale working memory, which could be an effect of the trauma or pre-existing differences.

The first step was to compare verbal learning and memory performance between patients and healthy controls. Given that learning performance at both time-points T1 and T2 was significantly different between groups, we controlled for learning in all memory analyses (Elliott et al., 2014). ANCOVAs (controlling for age and learning) were used to investigate group differences regarding episodic memory performance (recall and recognition performance as well as recall loss) at T1 and T2. Additionally, we used repeated measures ANCOVAs (controlling for learning and age) to investigate the influence of group on memory recall over time (immediate recall, 30-min recall and 1-week recall) as well as on memory recognition over time (30-min recognition and 1-week recognition) at T1 and T2. Furthermore, we examined the influence of acute-phase variables such as loss of consciousness and amnesia on verbal learning and memory performance at T1 and T2, using non-parametric *U*-tests. We also explored Pearson correlations between episodic memory performance in the PED and episodic memory performance at T1 or T2.

Second, we compared performance in tests of executive functions (working memory index, semantic fluency, interference control and flexibility) as well as parents’ memory rating between groups at T1 and T2 using ANCOVAs (controlling for age). Furthermore, we examined Pearson correlations between verbal learning, 1 week recall, recall loss and parents’ memory rating, executive functions as well as divided attention.

Third, we analyzed predictors for long-term memory and forgetting. We used linear regression analysis, introducing group, age, SES and pre-existing problems (representing pre-injury diagnosis and previous mTBI), verbal learning and executive functions (according to the size of correlations; for T1 we included flexibility and working memory; for T2 we included flexibility and divided attention). *R^2^* change was used as the indicator for the amount of individual variance explained by every new predictor. For both time-points, we used free recall after 1 week as well as recall loss between 30-min and 1-week recall as dependent variables.

## 3 Results

As illustrated in Table 1, our mTBI sample was mildly injured, with a mean GCS of 14.9, and one patient (1.6%) had intracranial injuries. A quarter of the patients (25%) were unconscious for a short period and 53% had post-traumatic amnesia. More children in the patient group had a previous mTBI (14%) than healthy controls (4%), whereas pre-injury diagnoses (learning problems and/or ADHD) were comparable between groups. Pre-existing problems (concatenating pre-injury diagnosis and previous mTBI) were significantly higher in children who had sustained mTBI compared to healthy controls [*X^2^*(1) = 5.26, *p* < 0.05, *φ* = −0.21]. The number of days between T1 and T2 did not differ between groups.

### 3.1 Memory performance 1 week after injury (T1)

The results of memory and executive function testing at T1 are provided in Table 2. At T1, children after mTBI showed a significantly reduced verbal learning and verbal recall after 1 week as well as a significantly elevated recall loss over time. In all other memory variables [immediate recall, 30-min recall and recognition performance (30 min and 1 week after learning)], patients performed comparably to healthy controls. As illustrated in Table 3, compared to patients who had not lost consciousness, patients reported to have been unconscious following injury had a significantly reduced 30-min recall as well as 1-week recall at T1, but a comparable verbal learning and recognition performance. Furthermore, in children after mTBI there were no group differences between those with and those without pre-existing problems (learning problems, ADHD, previous mTBI) regarding verbal learning, verbal recall and recognition performance at T1. Moreover, reported initial amnesia or vomiting did not influence verbal learning and memory performance at T1. Additionally, episodic memory recall in the PED did not correlate with 30-min or 1-week memory recall performance at T1. Parent-rated memory performance in everyday life was comparable between patients and healthy controls.

**Table 2:** Neuropsychological outcome in patients and healthy controls.

	Controls Mean (SD)	mTBI Mean (SD)	Test of significance (df)	*p*-value	Effect size
Episodic memory performance at T1 (1 week after injury; n_mTBI_: 64; n_controls_: 57)					
Verbal learning (raw) (maximum possible raw score: 68)	43.98 (7.93)	39.56 (9.86)	*F*(1,118) = 4.28	<0.05*	*η_p_^2^* = 0.04
Recall, immediate (raw) (maximum possible raw score: 17)	11.40 (2.49)	9.95 (3.30)	*F*(1,117) = 1.08	0.30	*η_p_^2^* = 0.01
Recall, 30-min (raw) (maximum possible raw score: 17)	11.35 (2.78)	10.03 (2.98)	*F*(1,117) = 0.52	0.47	*η_p_^2^* = 0.00
Recall, 1-week (raw) (maximum possible raw score: 17)	8.53 (3.13)	5.83 (3.34)	*F*(1,117) = 13.70	<0.001***	*η_p_^2^* = 0.11
Recognition^a^, 30-min (raw) (maximum possible raw score: 17)	14.68 (2.11)	13.58 (3.29)	*F*(1,117) = 0.69	0.41	*η_p_^2^* = 0.01
Recognition^a^, 1-week (raw) (maximum possible raw score: 17)	11.14 (4.20)	9.09 (5.15)	*F*(1,117) = 1.73	0.19	*η_p_^2^* = 0.02
Recall loss ^b^ (%)	24.93 (20.02)	43.23 (25.23)	*F*(1,117) = 17.57	<0.001***	*η_p_^2^* = 0.13
Memory rating (raw) (maximum possible raw score: 60)	18.23 (4.22)	18.80 (3.97)	*F*(1,117) = 0.98	0.324	*η_p_^2^* = 0.01
Executive and attentional functions at T1 (n_mTBI_: 64; n_controls_: 55)					
Interference control (SS)	11.54 (2.31)	10.43 (2.63)	*F*(1.117) = 5.28	<0.05*	*η_p_^2^* = 0.04
Working memory index (CS)	108.40 (12.26)	100.48 (15.47)	*F*(1,114) = 8.08	<0.01**	*η_p_^2^* = 0.07
Semantic fluency (SS)	14.02 (2.79)	12.22 (3.24)	*F*(1,118) = 9.19	<0.01**	*η_p_^2^* = 0.07
Flexibility (SS)	12.26 (2.72)	10.66 (3.32)	*F*(1,118) = 7.56	<0.01**	*η_p_^2^* = 0.06
Divided attention (T)	53.79 (11.12)	51.40 (10.92)	*F*(1,114) = 1.03	0.31	*η_p_^2^* = 0.01
Episodic memory performance at T2 (3–6 months after injury; n_mTBI_: 55; n_controls_: 51)					
Verbal learning (raw) (maximum possible raw score: 68)	45.88 (7.58)	40.09 (9.34)	*F*(1,104) = 8.67	<0.01**	*η_p_^2^* = 0.08
Recall, immediate (raw) (maximum possible raw score: 17)	11.49 (2.56)	10.02 (2.88)	*F*(1,103) = 0.00	0.95	*η_p_^2^* = 0.00
Recall, 30-min (raw) (maximum possible raw score: 17)	11.31 (3.16)	10.23 (2.74)	*F*(1,103) = 0.96	0.33	*η_p_^2^* = 0.01
Recall, 1-week (raw) (maximum possible raw score: 17)	7.39 (2.99)	5.07 (2.86)	*F*(1,102) = 6.69	<0.05*	*η_p_^2^* = 0.06
Recognition^a^, 30-min (raw) (maximum possible raw score: 17)	14.22 (3.16)	13.32 (4.04)	*F*(1,103) = 0.09	0.761	*η_p_^2^* = 0.00
Recognition^a^, 1-week (raw) (maximum possible raw score: 17)	10.27 (4.53)	7.35 (4.82)	*F*(1,102) = 3.66	0.059	*η_p_^2^* = 0.04
Recall loss ^b^ (%)	33.52 (24.82)	51.43 (23.49)	*F*(1,102) = 11.90	<0.01**	*η_p_^2^* = 0.10
Memory rating (raw)(maximum possible raw score: 60)	17.47 (3.06)	19.04 (4.44)	*F*(1,104) = 5.2	<0.05*	*η_p_^2^* = 0.05
Executive and attentional functions at T2 (n_mTBI_: 55; n_controls_: 50)					
Interference control (SS)	12.42 (1.71)	11.60 (2.18)	*F*(1,104) = 5.26	<0.05*	*η_p_^2^* = 0.05
Working memory index (CS)	112.21 (11.55)	102.29 (14.21)	*F*(1,105) = 13.93	<0.001***	*η_p_^2^* = 0.12
Semantic fluency (SS)	11.42 (2.89)	10.05 (2.57)	*F*(1,105) = 6.17	<0.05*	*η_p_^2^* = 0.06
Flexibility (SS)	11.27 (2.68)	9.59 (2.87)	*F*(1,105) = 10.89	<0.001**	*η_p_^2^* = 0.09
Divided attention (T)	54.84 (10.25)	55.27 (8.97)	*F*(1,104) = 0.03	0.855	*η_p_^2^* = 0.00

CS, Composite score; SS, Scaled score; T, T-value.^a^Recognition: no. of correctly identified minus false-positive answers.^b^Recall loss is computed as = 100* (recall 30 min after learning minus recall after 1 week)/30-min recall.*p < 0.05; **p < 0.01; ***p < 0.001.

**Table 3:** Comparison of verbal learning and memory recall at T1 between patients with and without reported initial loss of consciousness.

	Patients with loss of consciousness, *Mdn* (IQR)	Patients without loss of consciousness, *Mdn* (IQR)	Test statistics	*p*-value	Effect size (*r*)
T1	*n* = 16	*n* = 48			
Verbal learning	35 (29.50, 41.75)	40 (33, 47.75)	*U* = 266.5	0.068	−0.23
Immediate recall	8.50 (7, 11)	10.50 (8, 13)	*U = 262.5*	0.058	−0.24
30-min recall	8.50 (6, 10.75)	11 (8, 12.75)	*U* = 237	0.022*	−0.29
1-week recall	4.50 (3, 5.75)	5 (4, 9)	*U* = 253	0.041*	−0.26
Recall loss (%)	44.94 (38.13, 64.12)	40.66 (20, 62.50)	*U = 297.5*	0.180	−0.17
30-min recognition	13.50 (9.50, 15.75)	14 (12, 16)	*U = 301*	0.193	−0.16
1-week recognition	7.50 (4, 12)	10 (8, 13)	*U = 263*	0.060	−0.24

Mdn, Median; IQR, Inter quartile range. *p < 0.05.

**Figure 1:**
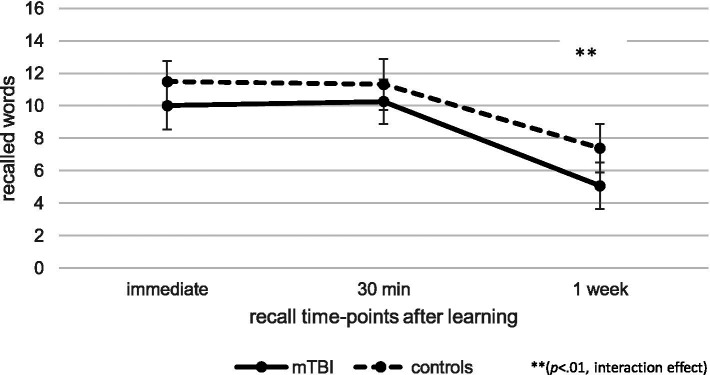
Comparison of verbal recall over time at T1 between patients after mild traumatic brain injury (mTBI) and healthy controls.

Regarding memory recall over time at T1, as illustrated in Figure 1, repeated measures ANCOVA (controlled for age and verbal learning) indicated no effect of time [*F*(1.56, 182.92) = 0.06, *p* = 0.90, *η_p_^2^* = 0.00], but a significant effect of group [*F*(1, 117) = 6.44, *p* < 0.05, *η_p_^2^* = 0.05] as well as a significant interaction effect between time and group [*F*(1.56, 182.92) = 9.27, *p* < 0.01, *η_p_^2^* = 0.07]. Post-hoc pairwise comparisons indicated that, compared to healthy controls, patients recalled fewer words 7 days after learning compared to immediately after learning (*p* < 0.001, *M* = − 3.49, 95% CI [−4.13, −2.85]) or 30 min after learning (*p* < 0.001, *M* = − 3.50, 95% CI [−4.06, −2.95]). When patients with pre-existing problems (pre-injury diagnosis and previous mTBI) as well as the one patient with intracranial injuries (mTBI *n* = 48; controls *n* = 49) were excluded, repeated measure ANCOVA regarding the influence of group and time on memory recall showed a significant interaction effect between time and group, without any effects of group or time. Recall loss was also significantly different between groups when the patients with pre-injury diagnosis (pre-injury mTBI or intracranial injury) were excluded. Thus, the results stayed the same, even when patients with pre-existing problems were excluded.

**Figure 2:**
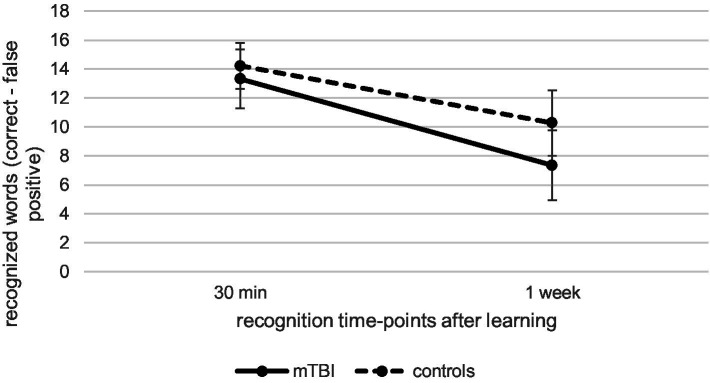
Comparison of verbal recognition over time at T1 between patients after mild traumatic brain injury (mTBI) and healthy controls.

Regarding recognition performance over time at T1, as illustrated in Figure 2, repeated measures ANCOVA (controlled for age and verbal learning) indicated a significant effect of time [*F*(1.00, 117.00) = 10.63, *p* < 0.01, *η_p_^2^* = 0.08], but no significant effect of group [*F*(1, 117) = 1.62, *p* = 0.21, *η_p_^2^* = 0.01] or interaction between time and group [*F*(1.00, 117.00) = 1.21, *p* = 0.27, *η_p_^2^* = 0.01]. This data indicates that recognition performance in patients and controls 1 week after learning was significantly reduced compared to 30-min recognition performance (*p* < 0.001, *M* = − 4.02, 95% CI [−4.63, −3.41]).

### 3.2 Memory performance 3–6 months after injury (T2)

Results of memory testing at T2 are also provided in Table 2. At T2, children after mTBI had a significantly reduced verbal learning performance and 1-week recall as well as a significantly elevated recall loss between the 30-min and 1-week recall compared to the healthy controls. In tests of all other memory variables (immediate recall, 30-min recall and recognition performance 30 min and 1 week after learning), patients performed comparably to healthy controls. Compared to controls, parent-rated memory performance in everyday life of children after mTBI was significantly reduced. However, loss of consciousness, acute amnesia after injury or pre-existing neurodevelopmental problems did not influence verbal memory performance in patients at T2.

**Figure 3:**
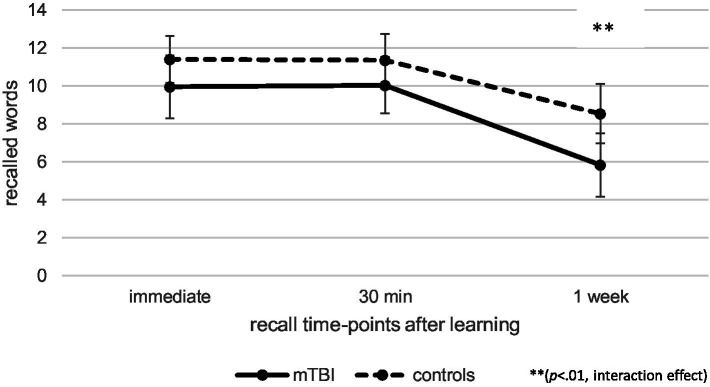
Comparison of verbal recall over time at T2 between patients after mild traumatic brain injury (mTBI) and healthy controls.

At T2, repeated measures ANCOVA (controlled for age and learning) indicated no effect of time [*F*(1.66, 169.00) = 0.13, *p* = 0.84, *η_p_^2^* = 0.00] or group [*F*(1, 102) = 0.66, *p* = 0.42, *η_p_^2^* = 0.01] regarding verbal recall over time, but there was a significant interaction effect between time and group [*F*(1.66, 169.00) = 8.73, *p* < 0.01, *η_p_^2^* = 0.08], as illustrated in Figure 3. Post-hoc pairwise comparisons indicated that patients after mTBI recalled fewer words 1 week after learning than immediately after learning (*p* < 0.001, *M* = − 4.50, 95% CI [−5.05, −3.96]) as well as 30 min after learning (*p* < 0.001, *M* = − 4.54, 95% CI [−5.15, −3.94]).

**Figure 4:**
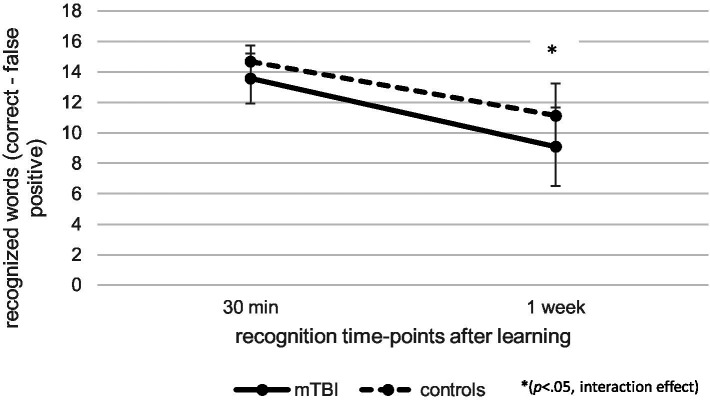
Comparison of verbal recognition over time at T2 between patients after mild traumatic brain injury (mTBI) and healthy controls.

Regarding recognition performance over time at T2, as illustrated in Figure 4, repeated measures ANCOVA (controlled for age and verbal learning) indicated a significant effect of time [*F*(1.00, 102.00) = 8.94, *p* < 0.01, *η_p_^2^* = 0.08] as well as a significant interaction effect between time and group [*F*(1.00, 102.00) = 5.82, *p* < 0.05, *η_p_^2^* = 0.05], but no effect of group [*F*(1, 102) = 1.29, *p* = 0.26, *η_p_^2^* = 0.01]. Post-hoc pairwise comparisons indicated that patients after mTBI recognized fewer words 1 week after learning than 30 min after learning (*p* < 0.001, *M* = − 4.96, 95% CI [−5.66, −4.25]). When patients with pre-existing problems (pre-injury diagnosis and previous mTBI) as well as the one patient with intracranial injuries were excluded, the results of the repeated measure ANCOVA regarding recall and recognition performance over time as well as recall loss did not change.

### 3.3 Executive functions and divided attention at T1 and T2

As shown in Table 2, at both time-points, children after mTBI had a significantly reduced performance in executive functions (interference control, working memory, semantic fluency and flexibility) compared to healthy controls. Groups did not differ regarding divided attention performance, either at T1 or at T2.

### 3.4 Associations between executive functions, divided attention, memory performance and memory rating in patients

Correlations between executive functions, divided attention and memory performance (verbal learning, 30 min recall, 1 week recall and recall loss) in patients after mTBI are shown in Table 4. At T1, there were moderate significant positive Pearson correlations between verbal learning and several executive functions (working memory, semantic fluency, flexibility) as well as between 30 min recall and the same executive functions. Additionally, there was a moderate positive correlation between 30 min recall and divided attention. There were no significant correlations between executive functions/divided attention and 1 week recall or recall loss. Furthermore, there were no significant correlations between parental memory rating and verbal learning or memory performance (30 min/1 week recall).

**Table 4:** Pearson correlations (*p*-value) between memory performance and executive/attentional functions as well as memory rating in children after mTBI.

	Interference control	Working memory	Semantic fluency	Flexibility	Divided attention	Parents’ memory rating
**T1**						
Verbal learning	0.224 (<0.05) *n* = 63	0.387 (<0.01)* n = 60	0.471 (<0.001)* n = 64	0.305 (<0.01)* n = 64	0.306 (<0.01) *n* = 60	−0.081 (0.262) *n* = 64
30-min recall	0.258 (0.021) *n* = 63	0.385 (0.001)* n = 60	0.416 (<0.001)* n = 64	0.387 (<0.001)* n = 64	0.401 (<0.001)* n = 60	−0.130 (0.153) *n* = 64
1-week recall	0.076 (0.277) *n* = 63	0.083 (0.265) *n* = 60	0.196 (0.061) *n* = 64	0.250 (<0.05) *n* = 64	0.163 (0.170) *n* = 60	−0.028 (0.414) *n* = 64
Recall loss	0.119 (0.177) *n* = 63	0.089 (0.249) *n* = 60	−0.015 (0.453) *n* = 64	−0.094 (0.230) *n* = 64	0.093 (0.240) *n* = 60	0.004 (0.486) *n* = 64
**T2**						
Verbal learning	0.367 (<0.01)* n = 55	0.436 (<0.001)* n = 56	0.265 (<0.05) *n* = 56	0.011 (0.468) *n* = 56	0.377 (<0.01)* n = 56	−0.227 (<0.05) *n* = 56
30-min recall	0.355 (<0.01)* n = 55	0.429 (<0.001)* n = 56	0.192 (0.078) *n* = 56	−0.006 (0.482) *n* = 56	0.451 (<0.001)* n = 56	−0.203 (0.064) *n* = 56
1-week recall	0.303 (<0.05) *n* = 54	0.300 (<0.05) *n* = 55	0.206 (0.065) *n* = 55	0.305 (0.012) *n* = 55	0.192 (0.080) *n* = 54	−0.103 (0.227) *n* = 55
Recall loss	−0.200 (0.074) *n* = 54	−0.145 (0.145) *n* = 55	−0.157 (0.125) *n* = 55	−0.357 (<0.01)* n = 55	−0.064 (0.322) *n* = 55	−0.024 (0.430) *n* = 55

*Bonferroni correction: *p* < 0.008.

At T2, within patients, there were significant moderate positive correlations between verbal learning and executive functions (interference control, working memory) as well as between verbal learning and divided attention. Moreover, there were significant moderate positive correlations between 30 min recall and executive functions (interference control and working memory) as well as between 30 min recall and divided attention. Additionally, there were moderate sized, but insignificant, positive correlations between 1 week recall and executive functions (interference control, working memory, flexibility). There was a significant negative correlation between recall loss and flexibility, while there were no significant correlations between parental memory rating and verbal memory performance (learning and recall 30 min/1 week).

### 3.5 Associations between executive functions, divided attention, memory performance, and memory rating in controls

Correlations between executive functions, divided attention and memory performance in healthy controls are shown in Table 5. At T1, there were significant positive correlations between working memory and verbal learning, working memory and 30 min recall as well as between working memory and 1 week recall. There were no significant correlations between executive functions/divided attention and recall loss or between verbal memory performance and parental memory rating. At T2, there were significant correlations between verbal fluency and verbal learning as well as between semantic fluency and verbal learning. Furthermore, there was a significant negative correlation between divided attention and recall loss. There were no significant correlations between memory performance (30 min recall or 1 week recall) and executive functions/divided attention. Furthermore, correlations between parental memory rating and memory performance were non-significant.

**Table 5:** Pearson correlations (*p*-value) between memory performance and executive/attentional functions as well as memory rating in healthy control children.

	Interference control	Working memory	Semantic fluency	Flexibility	Divided attention	Parents’ memory rating
**T1**						
Verbal learning	−0.007 (0.479) *n* = 57	0.322 (<0.01)* n = 57	0.239 (<0.05) *n* = 57	−0.091 (0.250) *n* = 57	0.301 (<0.05) *n* = 57	0.054 (0.347) *n* = 56
30-min recall	−0.066 (0.312) *n* = 57	0.409 (<0.001)* n = 57	0.160 (0.117) *n* = 57	−0.121 (0.185) *n* = 57	0.103 (0.222) *n* = 57	0.048 (0.362) *n* = 56
1-week recall	−0.033 (0.404) *n* = 57	0.448 (<0.001)* n = 57	0.158 (0.120) *n* = 57	−0.004 (0.488) *n* = 57	0.044 (0.372) *n* = 57	−0.019 (0.445) *n* = 56
Recall loss	−0.002 (0.494) *n* = 57	−0.170 (0.103) *n* = 57	−0.056 (0.340) *n* = 57	−0.072 (0.296) *n* = 57	0.094 (0.243) *n* = 57	0.170 (0.217) *n* = 56
**T2**						
Verbal learning	0.021 (0.442) *n* = 51	0.335 (<0.01)* n = 51	0.364 (<0.01)* n = 51	0.268 (<0.05) *n* = 51	0.263 (<0.05) *n* = 50	−0.116 (0.210) *n* = 50
30-min recall	0.011 (0.468) *n* = 51	0.271 (<0.05) *n* = 51	0.196 (0.084) *n* = 51	0.187 (0.094) *n* = 51	0.138 (0.170) *n* = 50	−0.061 (0.336) *n* = 50
1-week recall	0.172 (0.114) *n* = 51	0.227 (0.054) *n* = 51	0.214 (0.065) *n* = 51	0.066 (0.322) *n* = 51	0.310 (<0.05) *n* = 50	−0.249 (<0.05) *n* = 50
Recall loss	−0.209 (0.070) *n* = 51	−0.027 (0.426) *N* = 51	−0.055 (0.352) *n* = 51	0.033 (0.408) *n* = 51	−0.339 (<0.01)* N = 50	0.269 (<0.05) *N* = 50

^*^Bonferroni correction: *p* < 0.008.

### 3.6 Predictors of delayed recall performance after 1 week and recall loss at T1 and T2

The results of the regression analysis of T1 are illustrated in Table 6. Linear regression analysis indicated that group (*β* = 0.29, *p* < 0.001) and verbal learning (*β* = 0.44, *p* < 0.001) predicted delayed recall after 1 week [*F*(7,109) = 8.72; *p* < 0.001; *R^2^* = 0.36], but age, SES, pre-existing problems, and executive functions (flexibility and working memory) were not predictive. Regarding recall loss [*F*(7, 109) = 3.81; *p* < 0.001; *R^2^* = 0.20], group was the only significant predictor (*β* = −0.40; *p* < 0.001).

**Table 6:** Regression analysis to predict 1 week recall and recall loss at T1.

	*B*	*SE*	*β*	CI (95%)	*t*	*p*
**Predictors of 1-week recall**						
Constant	−2.29	2.70		−7.64 to 3.06	−0.85	0.40
Group	2.02	0.59	0.29	0.85 to 3.18	3.42	<0.001
Age	−0.09	0.13	−0.06	−0.34 to 0.16	−0.72	0.47
SES	−0.09	0.11	−0.06	−0.30 to 0.13	−0.79	0.43
Pre-existing problems	−0.66	0.82	−0.07	−2.28 to 0.96	−0.81	0.42
Verbal learning	0.17	0.03	0.44	−0.10 to 0.23	5.00	<0.001
Flexibility	0.04	0.09	0.04	−0.15 to 0.23	0.44	0.66
Working memory	0.01	0.02	0.05	−0.03 to 0.06	0.56	0.58
**Predictors of recall loss**						
Constant	59.77	21.00		18.14 to 101.14	2.85	0.005
Group	−19.46	4.59	−0.40	−28.55 to −10.36	−4.24	<0.001
Age	0.79	0.98	0.07	−1.14 to 2.73	0.81	0.42
SES	0.36	0.85	0.04	−1.33 to 2.04	0.42	0.67
Pre-existing problems	3.65	6.35	0.05	−8.94 to 16.24	0.58	0.57
Verbal learning	−0.29	0.26	−0.11	−0.81 to 0.22	−1.13	0.26
Flexibility	−0.60	0.73	−0.08	−2.04 to 0.85	−0.82	0.41
Working memory	0.09	0.17	0.05	−0.25 to 0.42	0.51	0.61

B, unstandardized beta coefficient; SE, standard error of B; β, standardized beta value; CI, confidence interval; SES, socioeconomic status. *p < 0.05.

Results of the regression analysis for T2 are summarized in Table 7. Similarly to T1, linear regression analysis indicated that group (*β* = 0.19, *p* < 0.05) and verbal learning (*β* = 0.47, *p* < 0.001) predicted delayed recall after 1 week [*F*(7, 97) = 8.69; *p* < 0.001; *R^2^* = 0.39]. Regarding recall loss [*F*(7, 97) = 3.77; *p* < 0.01; *R^2^* = 0.21], group (*β* = −0.31; *p* < 0.01) was the only significant predictor.

**Table 7:** Regression analysis to predict 1 week recall and recall loss at T2.

	*B*	*SE*	*β*	CI (95%)	*t*	*p*
**Predictors of 1-week recall**						
Constant	−4.38	2.55		−9.44 to 0.68	−1.72	0.09
Group	1.20	0.56	0.19	0.08 to 2.32	2.13	<0.05
Age	−0.06	0.12	−0.04	−0.29 to 0.18	−0.48	0.63
SES	−0.02	0.10	−0.02	−0.22 to 0.17	−0.23	0.82
Pre-existing problems	−0.29	0.78	−0.03	−1.84 to 1.25	−0.38	0.71
Verbal learning	0.16	0.04	0.47	0.10 to 0.23	4.96	<0.001
Flexibility	0.12	0.09	0.11	−0.06 to 0.31	1.34	0.19
Divided attention	0.03	0.03	0.08	−0.03 to 0.08	0.95	0.35
**Predictors of recall loss**						
Constant	89.18	23.32		42.89 to 135.46	3.82	<0.001
Group	−15.46	5.15	−0.31	−25.67 to −5.25	−3.00	<0.01
Age	2.15	1.08	0.20	0.02 to 4.29	2.00	0.05
SES	0.03	0.89	0.00	−1.74 to 1.79	0.03	0.98
Pre-existing problems	2.69	7.12	0.04	−11.44 to 16.82	0.38	0.71
Verbal learning	−0.42	0.30	−0.15	−1.02 to 0.17	−1.41	0.16
Flexibility	−0.87	0.84	−0.10	−2.54 to 0.81	−1.03	0.31
Divided attention	−0.38	0.26	−0.14	−0.84 to 0.14	−1.44	0.15

B, unstandardized beta coefficient; SE, standard error of B; β, standardized beta value; CI, confidence interval; SES, socioeconomic status. *p < 0.05.

## 4 Discussion

Interest in cognitive outcome after pediatric mTBI is increasing, and it has been assumed that pediatric mTBI is associated with a benign long-term cognitive outcome, without any lasting impairments (Babikian and Asarnow, 2009). Although we know from our clinical experience that memory impairments after mTBI are regularly reported by parents, few studies have focused on and been able to objectively assess episodic memory impairments after pediatric mTBI (Robertson-Benta et al., 2023), while other studies have found no impairments of verbal episodic memory after pediatric mTBI (Rieger et al., 2013; Studer et al., 2014). Our current study illustrates that, compared to healthy controls, children after mTBI had a comparable episodic memory performance in standardized testing 30 min after learning; however, 1 week after learning, they recalled significantly fewer words and showed a significantly greater recall loss over time. Thus, post-acutely as well as in the early chronic phase 3–6 months after injury, we found an elevated recall loss over time, demonstrating accelerated long-term forgetting in children after mTBI. The results stayed the same even when we excluded patients with pre-existing problems (learning problems, ADHD or previous mTBI) as well as the one patient with intracranial injuries. Thus, the effects are not explained as being due to patients having pre-existing problems or (in one case) intracranial injuries. The finding of a reduced delayed recall performance 1 week after learning, even 3–6 months after injury, is interesting, not only because our patient sample was very mildly injured, with a mean GCS of 14.9, but also because previous studies reported few, if any, neurocognitive impairments in patients after mTBI (Babikian and Asarnow, 2009).

Similar to the group differences in delayed memory performance at T2, parents of children after mTBI also reported more memory problems in daily life compared to parents of healthy controls. Even though 1-week recall performance was not significantly associated with parent-reported memory performance in daily life, we assume that delayed verbal memory performance is an ecologically valid and sensitive memory measure reflecting subtle memory impairments that would be missed by standardized testing focusing on 30-min recall. Furthermore, there are also other studies with adult patients indicating rather low associations between self-reported symptoms and cognitive testing: For example, in a study with patients after mTBI, changes in self-reported cognitive symptoms were not associated with changes in cognitive performance (Stenberg et al., 2020). Furthermore, there was no association between self-reported subjective memory functioning and memory performance after minor stroke (Geurts et al., 2019). Additionally, parents may not be able to perceive everyday memory problems as accurately as their children (Lineweaver et al., 2022), implying that future studies should include as well children’s self-rating about memory performance in everyday life.

Besides impairments in recall over time, we also found interaction effects between time and group regarding recognition performance 3–6 months after injury. This result implies that, after mTBI, not only recall performance, but also recognition performance after 1 week was reduced compared to 30-min recognition. Since the definition of ALF implies normal learning and memory recall performance in standardized memory testing (Elliott et al., 2014), it is assumed that ALF represents a disruption of memory storage rather than an acquisition deficit (Blake et al., 2000; Hoefeijzers et al., 2013; Mameniskiene et al., 2020). Given that both recall and recognition performance over time were impaired in children after mTBI, we interpreted our data in terms of a consolidation impairment, because reduced retrieval access should not lead to impaired recognition performance. Furthermore, if the reduced recall after 1 week is a consequence of an access impairment, we would expect executive functions to be predictive of 1-week recall or forgetting, which was not the case at either T1 or T2.

Nevertheless, although executive functions were not predictive for forgetting, our data illustrate that, in patients, several executive functions (interference control, working memory and flexibility) were associated with delayed memory performance 3–6 months after injury, which was not observable in healthy controls. Although these associations were not statistically significant (due to Bonferroni adapted significance values), they had a moderate effect size. This leads us to speculate that patients needed more executive resources to accomplish a comparable memory performance to that of controls, possibly to compensate for subtle executive impairments. Although patients’ performance in executive functions was within the average range, children after mTBI showed reduced executive functions compared to healthy controls, at both time-points. Thus, it could be that patients had subtle executive impairments for which they needed to compensate. Similar compensation processes have already been reported from neuroimaging studies illustrating that children after mTBI had a different task-related cognitive activation, despite comparable cognitive performance to controls (Rausa et al., 2020). For example, Westfall et al. (2015) reported that, up to 1 year after the mTBI, pediatric patients showed increased working memory task-related brain activation in frontotemporal regions, despite comparable cognitive performance to healthy controls. According to the authors, this reflected compensatory brain activation in patients to achieve an average working memory performance (Westfall et al., 2015).

Additionally, given that learning performance correlated significantly with executive performance, especially in patients, we assume that better executive functions lead to a more structured encoding, which may have positively influenced recall performance 1 week after learning. Similarly, and interestingly, there is evidence that adult patients after mTBI underutilize semantic clustering strategies during verbal learning, leading to a reduced verbal memory recall in standardized tests (Geary et al., 2011; Broadway et al., 2019). Unfortunately, our memory test did not allow us to investigate semantic categorization during encoding because it included semantically unrelated words. However, our results imply an association between executive functions and verbal learning that could have negatively influenced delayed memory recall.

Given that learning performance and group were the only predictive factors for recall performance after 1 week, encoding seems to be important for delayed memory performance, despite earlier findings that ALF is not associated with an acquisition impairment (Blake et al., 2000; Hoefeijzers et al., 2013). Our results indicate that ALF could be related to a consolidation impairment in children after mTBI although encoding and retrieval could still have influenced outcome. Additionally, recent evidence from a study in healthy adults suggests that wakeful rest, a methodologically accepted approach to promote and study consolidation, had a positive effect on recall performance, but not on recognition (Millar and Balota, 2022), possibly implying that consolidation processes may have different effects on recall and recognition performance. Thus, future studies are needed to investigate the role of encoding as well as the complex interplay between acquisition, storage and retrieval on delayed memory performance, to investigate the processes underlying ALF (Wagner et al., 2005; Shimamura, 2011; Levy, 2012). Future studies should also investigate the influence of sleep on delayed memory performance. There is mounting evidence of an association between slow-wave sleep and memory consolidation in adults (Diekelmann and Born, 2010; Wilhelm et al., 2012). We therefore assume that delayed memory performance after 1 week could also be related to other microstructural sleep factors such as sleep spindles. Recent studies have illustrated that sleep spindles are associated with sleep-dependent memory consolidation in healthy children (Hahn et al., 2019; Hoedlmoser, 2020), suggesting that sleep spindles could be a proxy measure for quality of hippocampal-neocortical memory transformation. Interestingly, so far, no study has investigated the influence of microstructural sleep factors and memory consolidation over time in children, while the few studies that investigated the influence of sleep on delayed memory performance in adult patients reported mixed results (Atherton et al., 2014, 2016; Hoefeijzers et al., 2015). It seems that sleep does not always improve memory consolidation overnight, especially in patients with epilepsy, implying that epileptiform activity at night may confound consolidation (Galer et al., 2015). Thus, the influence of sleep on memory consolidation needs further investigation, especially in non-epileptic patients.

To the best of our knowledge, this is the first study investigating delayed verbal recall and recognition after 1 week in children after mTBI. Similar to findings in children after severe TBI (Lah et al., 2017), our results imply that patients with mTBI have a reduced 1-week recall and elevated forgetting over time. Thus, our results suggest that patients without epilepsy can also have elevated forgetting, as recently concluded in a systematic review of ALF in neuropediatric patients (Stähli et al., 2022). Given that these children would have been missed by a standardized memory test, our results suggest that delayed recall measures should be included in clinical neuropsychological evaluation of patients with neurological disease or acquired injuries.

This study has the following limitations: First, we did not include performance validity testing and thus might have overestimated the observed effects. Second, we assessed mood and anxiety only at the time of inclusion in the study, but not during the study and could thus have overlooked patients with a comorbid depressive or anxious state that developed during the weeks after injury. Third, due to the high number of patients who did not participate (around 73%), our sample may not be representative of the general mTBI population. Fourth, we did not include another injury group to control for a general injury effect (Babikian et al., 2011). Thus, our results might not only reflect the effects of mTBI, but could also illustrate premorbid individual differences between children.

## 5 Conclusion

The results of this study imply that children after mTBI show ALF, even 3–6 months after injury, possibly related to subtle impairments in memory consolidation. Given that standardized memory assessments do not include delayed memory retrieval 1 week after learning, we suggest that it be integrated in clinical evaluations of episodic memory, including in patients without seizures.

## Data availability statement

The raw data supporting the conclusions of this article will be made available by the authors, without undue reservation.

## Ethics statement

The studies involving humans were approved by Ethics Committee of the University Children’s Hospital in Bern and by the Bernese Cantonal Ethics Committee (number: 2020-00596). The studies were conducted in accordance with the local legislation and institutional requirements. Written informed consent for participation in this study was provided by the participants’ legal guardians/next of kin.

## Author contributions

KL: Conceptualization, Formal analysis, Methodology, Supervision, Writing – original draft, Writing – review & editing. ZA: Writing – original draft, Writing – review & editing, Project administration, Visualization, Data curation. FR: Project administration, Writing – original draft, Writing – review & editing, Methodology. KW: Writing – original draft, Writing – review & editing, Formal analysis. SB: Formal analysis, Writing – original draft, Writing – review & editing, Conceptualization, Supervision, Methodology. MS: Conceptualization, Supervision, Writing – original draft, Writing – review & editing, Formal analysis, Funding acquisition, Investigation, Methodology, Project administration, Visualization.
